# Genome-Wide Identification and Evolutionary Analysis of *Ionotropic Receptors* Gene Family: Insights into Olfaction Ability Evolution and Antennal Expression Patterns in *Oratosquilla oratoria*

**DOI:** 10.3390/ani15060852

**Published:** 2025-03-16

**Authors:** Wen-Qi Yang, Ge Ding, Lin-Lin Wang, Chi-Jie Yin, Hai-Yue Wu, Hua-Bin Zhang, Qiu-Ning Liu, Sen-Hao Jiang, Bo-Ping Tang, Gang Wang, Dai-Zhen Zhang

**Affiliations:** 1 Jiangsu Provincial Key Laboratory of Coastal Wetland Bioresources and Environmental Protection, Jiangsu Key Laboratory for Bioresources of Saline Soils, Jiangsu Synthetic Innovation Center for Coastal Bio-Agriculture, Yancheng Teachers University, Yancheng 224051, China; wqy20010929@163.com (W.-Q.Y.); 13763425231@163.com (L.-L.W.); yinchijie@outlook.com (C.-J.Y.); wuhaiyue929@outlook.com (H.-Y.W.); zhanghb@yctu.edu.cn (H.-B.Z.); liuqn@yctu.edu.cn (Q.-N.L.); longdance@sina.com (S.-H.J.); boptang@163.com (B.-P.T.); 2 College of Fisheries and Life Science, Shanghai Ocean University, Shanghai 201306, China; 3 Chemical and Biological Engineering College, Yancheng Institute of Technology, Yancheng 224003, China; dinggeyc@163.com

**Keywords:** *Oratosquilla oratoria*, olfaction, *Ionotropic Receptors*, genome-wide identification, fluorescence in situ hybridization

## Abstract

Olfaction plays a crucial role in crustaceans for essential activities such as foraging and predator evasion. Among the components involved in olfactory perception, *Ionotropic Receptors* (*IRs*) are particularly important. *Oratosquilla oratoria*, a perennial crustacean of substantial economic and ecological value, serves as an ideal model for studying olfactory mechanisms. Identifying the *IR* chemosensory genes in *O. oratoria* enhances our understanding of its olfactory recognition system. Based on the whole-genome data of *O. oratoria*, we identified and analyzed 50 members of the *IR* gene family (*OratIRs*) through bioinformatics approaches. These genes were classified into subfamilies of co-receptor *IRs* and tuning *IRs*. The physicochemical properties of the encoded proteins exhibit marked variability, indicating distinct roles. The motif types and conserved domains among these subfamilies display certain similarities, but their gene structures differ markedly. Furthermore, we found that *OratIR25a*, *OratIR07629*, and *OratIR14286* are key nodes in protein–protein interaction networks, coordinating organisms’ responses to signals like temperature and acids. We utilized fluorescence in situ hybridization (FISH) to find that *OratIR75-1* and *OratIR8a* demonstrated robust expression signals in the antennae of the *O. oratoria*. These findings lay a foundation for further investigations and elucidate the functional roles of olfactory receptor genes in crustaceans.

## Simple Summary

*Ionotropic Receptors* (*IRs*) are essential chemical receptor genes that play a pivotal role in olfactory perception among crustaceans, substantially influencing behaviors such as foraging, mating, and predator avoidance. *Oratosquilla oratoria* is an omnivorous species known for its acute sense of smell. In this study, we employed bioinformatics methods to identify and analyze the members of the *IR* gene family in *O. oratoria* to elucidate the potential functions of *IRs* in olfactory perception within this species. The analysis encompassed various aspects including physical and chemical properties, chromosomal locations, structural features, expression characteristics, and phylogenetic studies both within and between species. Our findings identified 50 *IRs* in *O. oratoria*, which were categorized into co-receptor *IRs* and tuning *IRs*. Co-receptor *IRs* exhibited a high degree of conservation across three crustacean species. In contrast, tuning *IRs* displayed several tandem repeat genes. Fluorescence in situ hybridization (FISH) revealed that *OratIR75-1* was co-expressed with *OratIR8a* in the antenna tissues of *O. oratoria*, indicating its important role in olfactory processes. These results suggest that *IRs* are integral to the olfactory recognition mechanisms in *O. oratoria*. This research provides a scientific foundation for further investigations into the functional roles of *IRs* within this species.

## 1. Introduction

Chemical sensation constitutes a vital sensory system for crustaceans [1,2,3]. Over an extended evolutionary period, crustaceans have developed a sophisticated array of chemical sensory systems capable of detecting external environmental information [4,5,6]. By employing highly specific and sensitive chemical receptors for chemical communication, they can adapt to environmental pressures, seek advantages, evade threats, and ensure both individual development and population reproduction [7,8,9]. Olfaction, as a chemical sense, is widely understood as the ability to distinguish odors, capable of detecting a large number of small, light volatile compounds (i.e., odors), allowing organisms to gather rich information about their chemical environment and identify substances to be sought or avoided [6,10,11,12]. Consequently, olfaction ranks among the most important chemical senses in crustaceans.

*Ionotropic Receptors* (*IRs*) constitute a family of ion channels derived from *ionotropic glutamate receptors* (*iGluRs*) and belong to a variant subfamily of *iGluRs*. They were initially identified in *Drosophila melanogaster* as crucial sensors for detecting environmental and intercellular chemical signals [13,14,15,16]. Both *IRs* and *iGluRs* exhibit similar molecular architectures, characterized by the presence of a conserved ligand-gated ion channel domain [15,16,17,18], including the extracellular N terminus, intracellular C terminus, and a ligand-binding domain (LBD) composed of S1 and S2 segments, along with the ion channel domain (ICD). Notably, the ion channel domain is the most conserved region across both *IRs* and *iGluRs*, consisting of three transmembrane domains (TM1–TM3) along with an ion channel pore (P), indicating that *IRs* may have retained their capacity for ion conduction [14]. However, unlike *iGluRs*, *IRs* do not possess a differentiated extracellular amino-terminal domain (ATD); instead, most *IRs* feature only a short N-terminal region preceding the S1 segment of the LBD [19].

*iGluRs*, as a conserved family of ligand-gated ion channels, primarily encompass NMDA receptors and non-NMDA receptors (AMPA and Kainate receptors) [11]. *IRs* evolved from *iGluRs* and diversified into various lineages throughout the evolutionary process. They can be categorized into two primary subfamilies: co-receptor *IRs* and tuning *IRs* [15,20,21,22,23]. The co-receptor *IRs* comprise four types: *IR25a*, *IR8a*, *IR93a*, and *IR76b*, which are co-expressed with other *IRs* [13,24]. Notably, both *IR25a* and *IR8a* exhibit distinctive characteristics by retaining the ATD of *iGluRs* absent in most tuning *IRs*, which is essential for the formation of functional receptor channels [14,15]. Tuning *IRs* display considerable variability among different species; in some species, they demonstrate enhanced specificity. These receptors form functional heterotetrameric channels through interactions with co-receptor *IRs*, thereby determining the specific binding characteristics of the receptor [13,25]. In addition to their role as chemoreceptors, *IRs* also participate in detecting temperature and humidity signals—showcasing a remarkable adaptability to environmental stimuli [26,27,28,29]. This structural and functional diversity enables *IRs* to fulfill an irreplaceable role in organisms’ adaptation to their environments.

Substantial progress has been made in elucidating the functions of *ionotropic receptor* (*IR*) genes in model organisms such as *Drosophila*. However, research efforts dedicated to *IRs* in crustaceans remain relatively limited. Studies on *IRs* across various crustacean species have yielded diverse results. In *Daphnia*, *IR25a* and *IR93a* were identified, along with a large number of divergent *IRs* [15]. In the terrestrial hermit crab *Coenobita clypeatus*, 20 candidate *IR* genes were discovered, including the conserved *IR25a* and *IR93a* [30]. In *Panulirus argus*, 108 *IRs* were identified, with higher expression levels observed in the antennular lateral flagella [31]. Transcriptomic analysis of olfactory sensory neurons (OSNs) in this species further revealed that OSNs express co-receptor *IRs* such as *IR25a* and *IR93a*, along with 9–53 tuning *IRs*, which vary in abundance [32]. Similarly, in *Panulirus ornatus*, tissue analysis showed 70 upregulated *IR* isoforms in aesthetasc-bearing regions of the antennules, including co-receptors (*IR25a* and *IR93a*) and divergent receptors (*IR4*, *IR7*, and *IR21a*) [33]. In *Eriocheir japonica sinensis*, 33 *EsIRs* were identified, highlighting their role as key odorant receptors with a specific evolutionary trend [34]. Decapod crustaceans, including *P. argus*, *Homarus americanus*, *Procambarus clarkii*, and *Callinectes sapidus*, express a high number of *IRs* ranging from 100 to 250. These *IRs* exhibit varying degrees of phylogenetic conservation and are more highly expressed in the lateral flagellum (LF) than in the dactyls [35]. Given the advancements in understanding *IR* expressions and characteristics in the aforementioned crustaceans, the paucity of research on *IRs* in Stomatopoda becomes particularly evident. This underscores the urgent need for further investigation into their functional roles and evolutionary significance.

*Oratosquilla oratoria*, commonly known as the mantis shrimp, or mantis prawn, belongs to the phylum Arthropoda, class Crustacea, order Stomatopoda, family Squillidae, and genus *Oratosquilla*. This perennial marine crustacean holds substantial economic value [36,37,38,39,40,41]. *O. oratoria* is a carnivorous species that primarily preys on small invertebrates. As a dominant member of the order Stomatopoda, it is widely distributed in the nearshore waters of the Northwest Pacific [42,43]. Valued for its tender meat and high protein content as well as its notable nutritional and pharmacological benefits, it enjoys considerable consumer preference [41,44]. However, due to overfishing and environmental degradation, *O. oratoria* resources are currently facing a severe decline while their economic value continues to rise [45]. To safeguard existing populations while addressing increasing consumption demands, strategies aimed at enhancing *O. oratoria* yield through research into its feeding-related chemoreception genes are essential for improving feeding efficiency. Comprehensive studies on the olfactory system of *O. oratoria* facilitate an understanding of how this species identifies informational substances when foraging and locating mates; they also elucidate mechanisms underlying chemical communication and perception. A substantial number of olfactory-related genes provide a foundation for investigating molecular mechanisms associated with these processes. Therefore, identifying and characterizing *IR* genes is crucial for studying gene function and revealing the intricacies of olfactory recognition.

This study identified the homologous genes within the *IR* gene family of *O. oratoria* utilizing whole-genome data and bioinformatics methodologies. The research systematically analyzed their physicochemical properties, chromosomal localization, phylogenetic evolution, and sequence characteristics. Furthermore, fluorescence in situ hybridization (FISH) was employed to localize the expression of two genes within this subfamily in the antennae and identify important regulatory genes of the *IR* gene family through protein–protein interaction mining. The research achievements will provide a theoretical foundation for a deeper understanding of the functions and mechanisms associated with the *IRs* of *O. oratoria*.

## 2. Materials and Methods

### 2.1. Sample Collection and Preparation

*O. oratoria* samples were collected from the Yellow Sea at Huangsha Port in Yancheng City, Jiangsu Province (33°44′ N, 120°24′ E). The selected specimens exhibited a body length ranging from 141 to 144 mm and a body width ranging from 30 to 32 mm. These samples were subsequently housed temporarily in the aquarium of the Jiangsu Provincial Key Laboratory of Saline Soil Biological Resources. A healthy specimen of *O. oratoria* was selected for further analysis; its antenna tissue was fixed using an in situ hybridization fixative to facilitate subsequent fluorescence in situ hybridization with paraffin sections.

### 2.2. Genomic Data Acquisition

Genomic data for *O. oratoria* [46] (GCA_046742065.1) were obtained from the Jiangsu Provincial Key Laboratory of Coastal Wetland Bioresources and Environmental Protection. The genomic data of Arthropoda utilized in this study were obtained from the National Center for Biotechnology Information (NCBI) database (https://www.ncbi.nlm.nih.gov/, accessed on 6 June 2024). The NCBI accession numbers for *Litopenaeus vannamei* [47] and *Macrobrachium nipponense* [48] are GCA_003789085.1 and GCA_015104395.1, respectively. The data for each downloaded species were standardized to ensure complete protein sequence information was available.

### 2.3. Sequence Search and Chromosomal Mapping Analysis of the IR Gene Family in O. oratoria

*IR* and *iGluR* genes were identified based on the genomic data of *O. oratoria*. (i) Construction of a Reference Sequence Library: The *IR* and *iGluR* protein sequences were obtained from publicly available datasets as well as the NCBI database (Table S1), which served as query sequences to establish the reference sequence library [15,31,35]. (ii) Homology Alignment: Using BLAST [49] software (version 2.13.0), we aligned the protein sequences of *O. oratoria* with the reference sequence database, setting an E-value threshold of ≤1 × 10^−9^ to identify candidate protein sequences corresponding to *IR* and *iGluR* within the *O. oratoria* protein database. (iii) Domain Alignment: Hidden Markov Model (HMM) profiles of PF00060, PF10613, and PF01094 were downloaded from the PFAM database (http://pfam.xfam.org, accessed on 7 June 2024) and used as query library files. We employed HMMER software (version 3.0) (http://hmmer.org, accessed on 7 June 2024) to search for specific domains associated with the *IR* gene family in these candidate protein sequences. By identifying sequences containing the Pfam domain PF00060, we predicted the ICD domain (including P, TM1–TM3) and the S2 region of the LBD. Additionally, we utilized PF10613 to predict the S1 region of the LBD [15,35]. Sequences that simultaneously contain the PF00060, PF10613, and PF01094 domains are *iGluR* gene family sequences. Sequences lacking these characteristic domains were filtered out to yield a final set of *IR* and *iGluR* gene family sequences. In addition, the *IR* and *iGluR* gene families of *L. vannamei* and *M. nipponense* were identified using the aforementioned methods. The identification of *iGluRs* was exclusively for constructing the phylogenetic tree in Section 2.5, which aimed to elucidate the evolutionary origins and relationships of the *IR* gene family.

All examined *IR* and *iGluR* genes were categorized into three groups based on their characteristics [31,35]: (i) the *iGluRs* group, which includes NMDA and non-NMDA (AMPA, Kainate); (ii) the co-receptor *IRs* subfamily; and (iii) the tuning *IRs* subfamily. Sequences from *O. oratoria* were assigned the following prefix: *Orat* (*O. oratoria*). The *IR* sequences in *O. oratoria* were designated with the prefix *OratIR*, followed by the original gene sequence number (e.g., *OratIR02114*, *OratIR03796*). Homologous sequences were named according to their corresponding *IR* homologs (e.g., *OratIR25a*, *OratIR8a*). In cases where a sequence within a species had multiple homologs, the suffixes ‘-1’, ‘-2’, etc., were appended to each homolog. NMDA *iGluRs* received designations beginning with the prefix *OratNMDAr*, followed by the suffixes ‘-1’, ‘-2’, etc., while non-NMDA *iGluRs* were designated using the prefix *OratGluR* with similar suffixes. Each *IR* gene in *O. oratoria* was designated as *OratIRs*. This nomenclature also applies to newly identified *IR* and *iGluR* genes in *L. vannamei* and *M. nipponense*.

To determine the distribution of *OratIR* genes across the chromosomes, we utilized the genomic annotation of *O. oratoria* to identify the chromosomal positions of these genes. The Gene Density Profile tool in TBtools (version 1.108) [50] was employed to generate gene density files, while the Gene Location Visualizer (Advanced) was used to visualize the locations of *OratIRs* on the chromosomes, effectively marking each gene’s position to elucidate their distribution throughout the chromosomal landscape.

### 2.4. Physicochemical Properties and Subcellular Localization Analysis of the IR Gene Family

The identified *IR* genes were analyzed using the ProtParam program available on the ExPASy service platform [51] (https://web.expasy.org/protparam/, accessed on 10 June 2024). This analysis calculated various physicochemical properties of the proteins, including amino acid length (aa), molecular weight (MW), theoretical isoelectric point (pI), instability index, aliphatic index, and grand average of hydropathicity (GRAVY). Furthermore, the subcellular localization of the *IR* genes was predicted utilizing the ProtComp 9.0 program from the Softberry service platform (http://www.softberry.com/, accessed on 11 June 2024).

### 2.5. Multiple Sequence Alignment and Phylogenetic Tree Construction Analysis of the IR Gene Family

To investigate the phylogenetic relationships among the members of the *IR* gene family in *O. oratoria*, as well as to explore the evolutionary and taxonomic relationships among various crustacean species, we constructed an intraspecific phylogenetic tree utilizing the *IR* and *iGluR* protein sequences derived from *O. oratoria*. Furthermore, interspecific phylogenetic analyses were conducted using *IR* and *iGluR* protein sequences from two additional species: *L. vannamei* and *M. nipponense*, with the *iGluR*/*IR25a*/*IR8a* branch serving as the root node [35]. Multiple sequence alignment of the *IR* gene family was performed using the MUSCLE tool (Multiple Protein Sequence Alignment) within MEGA11 software (version 6.0) [52]. Through the PhyML program in MEGA11, the maximum likelihood method (ML), incorporating 1000 bootstrap replicates, was employed to construct the phylogenetic tree, while iTOL [53] was utilized for visualization and enhancement of this tree.

### 2.6. Characterization of the IR Gene Family: Motif, Gene Structure, and Domain Prediction Analysis

To enhance the understanding of the function and evolution of members of the *IR* gene family, an analysis of conserved motifs, gene structures, and conserved domains can provide insights into their functional conservation and evolutionary changes. The identified *OratIR* gene family members were subjected to predictive analysis using the online tool MEME Suite [54] (https://meme-suite.org/meme/tools/meme, accessed on 13 June 2024). The parameters for this analysis were set as follows: minimum width: 30 bp, maximum width: 100 bp, and a maximum motif number of 8; all other parameters were maintained at their default settings. The Gene Structure Display Server (GSDS) [55] (https://gsds.gao-lab.org, accessed on 13 June 2024) was utilized to delineate the exon–intron structures of the *OratIR* genes, while TBtools was employed for visualizing these gene structures. The conserved domains of the *OratIR* gene family members were identified using the CD-search [56] tool on the NCBI official website (https://www.ncbi.nlm.nih.gov/Structure/bwrpsb/bwrpsb.cgi, accessed on 15 June 2024). The GOR4 website (https://npsa-prabi.ibcp.fr/cgi-bin/npsa_automat.pl?page=npsa_gor4.html, accessed on 16 June 2024) was used to predict the secondary structure of *OratIR* gene family members, and the SWISS-MODEL online platform (https://swissmodel.expasy.org/interactive, accessed on 18 June 2024) was utilized for protein 3D structure prediction.

### 2.7. Protein–Protein Interaction (PPI) Network Analysis of the IR Gene Family

To investigate and elucidate the interactions between proteins and to infer the regulatory roles of the *IR* gene family, *Drosophila* was selected as the reference species. The identified members of the *OratIR* gene family were utilized to ascertain protein interactions with other genes via the STRING protein interaction database (https://string-db.org/, accessed on 20 June 2024), with a maximum of 50 interactions displayed in the first layer. Following the acquisition of these interaction relationships, Cytoscape software (version 3.9.1) was employed to construct visual representations and analyze the interaction network.

### 2.8. Fluorescence In Situ Hybridization of the IR Gene Family

This experiment utilized paraffin sectioning and the SweAMI fluorescent in situ hybridization double staining technique. The in situ hybridization probes were designed by Servicebio, as listed in Table 1. The antennal tissue of *O. oratoria* was rinsed with phosphate-buffered saline (PBS) and subsequently immersed in an in situ hybridization fixative at 4 °C for a minimum of 12 h. Following fixation, the target area of the tissue was excised to approximately 3 mm thickness within a fume hood, dehydrated through a graded series of alcohols, cleared using xylene, and then embedded in paraffin. The paraffin-embedded blocks were sliced into sections measuring 4 μm thick, which were then spread out and baked in an oven at 62 °C for two hours. Following this, the sections were sequentially immersed in two changes of dewaxing solution for 15 min each. They were then treated with pure ethanol and a graded series of ethanol (85% and 75%) for 5 min each, before being soaked in DEPC water to complete the dewaxing process. Citrate buffer (pH 6.0) was utilized as a repair solution; the sections were placed in a repair box and heated in a water bath at 90 °C for 48 min to facilitate antigen retrieval. After natural cooling, the sections underwent digestion with proteinase K (20 μg/mL) at 37 °C for 5 min, followed by three washes with PBS for 5 min each. Subsequently, pre-hybridization solution was added, and the sections were incubated at 37 °C for one hour. The pre-hybridization solution was then discarded, after which a hybridization solution containing *OratIR8a*/*OratIR75-1* probes (Servicebio, Wuhan, China) was introduced at a concentration of 500 nm. The sections were hybridized overnight at 40 °C. After hybridization, the sections were washed again before adding hybridization solutions containing signal probe 1 labeled with FAM (488) and signal probe 2 labeled with Cy3 (dilution ratio of 1:200), followed by incubation at 42 °C for three hours. The sections underwent another washing step. Finally, a DAPI staining solution was applied to counterstain the nuclei. The stained sections were observed and imaged using a Nikon Eclipse ci upright fluorescence microscope.

### 2.9. RT-qPCR Validation of the Expression of Five OratIRs in O. oratoria

In this study, RNA isolater Total RNA Extraction Reagent (Vazyme, Nanjing, China) was used to extract RNA from the muscle and antennal tissues of *O. oratoria*. Subsequently, the integrity of the RNA was examined via 1% agarose gel electrophoresis, and its concentration and purity were measured using a NanoDrop 2000 (Thermo Fisher Scientific, Waltham, MA, USA). After the RNA had passed the quality assessment, reverse transcription was carried out using HisyGo RT Red SuperMix for qPCR (+gDNA Wiper) (Vazyme, Nanjing, China) to synthesize cDNA for subsequent RT-qPCR verification.

Specific RT-qPCR primers for five members of the *OratIRs* gene family were designed using Primer Premier 6.0 software (Table 2), and *β-actin* was selected as the reference gene. Finally, RT-qPCR was performed in a 10 μL reaction system, which consisted of 5 μL of 2 × SupRealQ Purple Universal SYBR qPCR Master Mix, 0.5 μL of forward primer, 0.5 μL of reverse primer, 2 μL of cDNA template, and 2 μL of ddH₂O. The reaction program was as follows: pre-denaturation at 95 °C for 30 s; followed by 40 cycles of denaturation at 95 °C for 10 s, annealing at 56 °C for 30 s, and extension at 72 °C for 30 s; and a melting curve analysis of 95 °C for 30 s, 60 °C for 1 min, and 95 °C for 10 s.

The relative expression levels of the genes were calculated using the 2^−ΔΔCT^ method [57]. Differences in gene expression between the two tissues were compared using *t*-tests. Visualization was performed using Prism software (version 5.01).

## 3. Results

### 3.1. Identification, Classification, and Chromosomal Distribution of the IR Gene Family in O. oratoria

Utilizing the Hidden Markov Model of Lig_chan (PF00060) and Lig_chan-Glu_bd (PF10613), in conjunction with BLASTp homologous alignment, we conducted a comprehensive search for *IR* protein sequences containing conserved PFAM domains. After removing duplicate sequences, we identified a total of 50 *IR* protein sequences. Among these, the *IRs* consist of 4 co-receptor *IRs* and 46 tuning *IRs*, designated according to their subfamily classification. In the genomes of the closely related species *L. vannamei* and *M. nipponense*, 28 and 74 *IR* genes were identified, respectively (Table S2).

**Figure 1:**
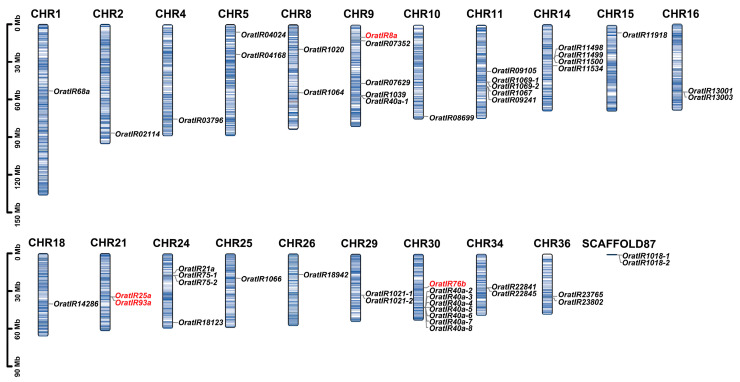
Chromosome location analysis of *OratIRs*. The black scale on the left represents the position, with column length representing chromosome size, and blue lines within the columns representing gene density on the chromosomes.

The chromosomal localization analysis of the *OratIR* gene family members (Figure 1) revealed that the 50 *OratIRs* are distributed across 20 distinct chromosomes and scaffold 87. The majority of these genes are situated in high-density regions, with varying numbers present on different chromosomes. Notably, chromosome 30 harbors a greater number of *IR* genes. The distribution patterns of *IR* subtypes differ among chromosomes. Co-receptor *IRs* are located on chromosomes 9, 21, and 30 while tuning *IRs* represent the most numerous category, dispersed across the remaining 19 chromosomes and on unassembled scaffold 87, excluding chromosome 21. Additionally, gene clustering was observed on chromosomes 11, 14, 24, and 30, as well as on scaffold 87. We propose that these clusters may have originated from tandem repeats, which is supported by their high density and clustering patterns. In the tuning *IRs*, the genes *OratIR1069-1* and *OratIR1069-2* cluster together, while *OratIR11498*, *OratIR11499*, and *OratIR11500*, like *OratIR75-1* and *OratIR75-2*, form a cluster. Furthermore, the genes *OratIR40a-2*, *OratIR40a-3*, *OratIR40a-4*, *OratIR40a-5*, and *OratIR40a-6* are clustered together, while *OratIR40a-7* and *OratIR40a-8* form another cluster. Additionally, the genes *OratIR1018-1* and *OratIR1018-2* exhibit clustering behavior as well. It was predicted that the distance between adjacent genes was less than 70 kb. Furthermore, their protein sequences exhibit a high degree of similarity, which further supports the hypothesis that these genes originated from tandem repeats (Table S3).

### 3.2. Physicochemical Properties and Subcellular Localization Analysis of the IR Gene Family

An analysis of the physicochemical properties and subcellular localization predictions of the *OratIR* gene family members was conducted, with results summarized in Table 3. The lengths of amino acids constituting the proteins of the *IR* gene family of *O. oratoria* vary significantly, ranging from 375 to 1882 amino acids (aa), while their molecular weights (MWs) span from 42.32 to 219.30 kDa. The average values are calculated at 726.34 aa and 81.90 kDa, respectively. We speculate that the substantial amino acid length variation in *OratIR* genes is likely due to gene duplications, followed by mutations altering gene regions and thus encoding lengths. The theoretical isoelectric points (pI) of the *OratIRs* range from acidic (4.0) to basic (9.4). Amino acids are classified based on their theoretical isoelectric points: those exceeding a pI of 7 are categorized as basic amino acids, whereas those below this threshold are classified as acidic amino acids. Among the analyzed set of 50 *IRs*, 16 consist predominantly of basic amino acids while the remaining 34 comprise acidic ones, and the acidic amino acids exhibit a higher average length and molecular weight compared to their basic counterparts. The instability index ranges from 27.98 to 87.04, while the aliphatic index spans from 68.18 to 106.92, and the grand average of hydropathicity (GRAVY) ranges from −0.902 to +0.229 overall; the proportion of hydrophobic proteins is 37%, while the proportion of hydrophilic proteins is 63%. According to the subcellular localization prediction results, 35 *OratIRs* encode proteins located in the plasma membrane, 6 genes localize in the endoplasmic reticulum, 5 genes reside in the golgi apparatus, 2 genes are situated in mitochondria, 1 gene is found in the vacuole, and 1 gene exists within extracellular space.

### 3.3. Interspecific and Intraspecific Phylogenetic Analysis of the IR Gene Family

**Figure 2:**
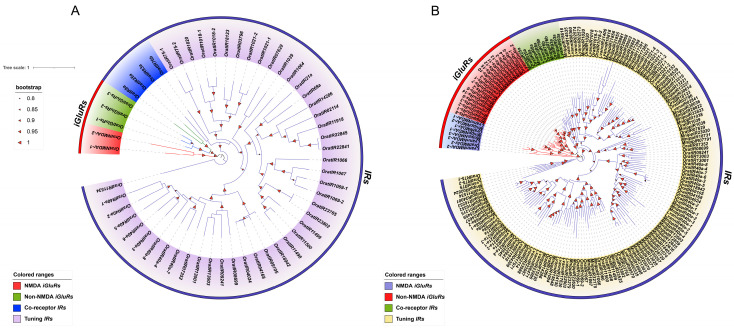
Phylogenetic tree. (**A**) Maximum-likelihood phylogenetic tree of *IRs* and *iGluRs* from *O. oratoria*. Different background colors represent different subfamilies: clades with NMDA *iGluRs* are colored red (lines are red); clades with non-NMDA *iGluRs* are colored green (lines are blue); clades with co-receptor *IRs* are colored blue (lines are green and purple); clades with tuning *IRs* are colored purple (lines are purple). (**B**) Maximum-likelihood phylogenetic tree of *IRs* and *iGluRs* from *O. oratoria*, *L. vannamei*, and *M. nipponense*. Clades with NMDA *iGluRs* are colored purple; clades with non-NMDA *iGluRs* are colored red; clades with co-receptor *IRs* are colored green; clades with tuning *IRs* are colored yellow. The outer circle in different colors represents subfamilies of *iGluRs* and *IRs*, with red indicating *iGluRs* and blue indicating *IRs* (lines: red for *iGluRs*, blue for *IRs*). *iGluRs*, *ionotropic glutamate receptors*; *IRs*, *ionotropic receptors*.

To investigate the phylogenetic relationships within the *IR* and *iGluR* gene families in *O. oratoria*, we constructed an intraspecific phylogenetic tree using the maximum likelihood method implemented in MEGA software (version 6.0). The results (Figure 2A) showed that the tree is divided into two primary branches, with genes of each subfamily clustering separately. Notably, co-receptor *IRs* exhibited a close relationship with *iGluRs*. The sister branch formed by *OratIR25a*, *OratIR8a*, and non-NMDA *iGluRs* suggested a closer evolutionary relationship. In contrast, tuning *IRs* displayed greater evolutionary diversity in the phylogenetic tree and could be further divided into three branches, including various conserved tuning *IRs*. These subclasses included arthropod-conserved tuning *IRs* (*IR21a*, *IR40a*, *IR68a*, *IR75*, and *IR1039*), crustacean-conserved tuning *IRs* (*IR1020*, *IR1064*, *IR1066*, and *IR1067*), and decapod-conserved tuning *IRs* (*IR1018*, *IR1021*, and *IR1069*). Additionally, other tuning *IRs* appeared to be specific to *O. oratoria*.

To further investigate the evolutionary relationships of *IR* genes among different species, we combined the *iGluR* and *IR* protein sequences of *O. oratoria*, *L. vannamei*, and *M. nipponense* to construct an interspecific phylogenetic tree (Figure 2B). The tree was divided into two major branches. Among different species, the *IR* and *iGluR* gene subtypes clustered in their respective branches due to sequence similarity, indicating that the *IR* gene family diverged prior to species divergence. Notably, the number of co-receptor *IRs* remained relatively conserved across species, whereas tuning *IRs* showed substantial interspecific variation. For example, *O. oratoria* has 46 tuning *IRs*, compared to 25 in *L. vannamei* and 70 in *M. nipponense*, suggesting lineage-specific expansion or contraction of this gene family. From the interspecific phylogenetic tree, it is evident that, in *L. vannamei*, co-receptor *IRs* exhibit a close relationship with *iGluRs*. The 25 tuning *IRs* in *L. vannamei* can be divided into three branches, similar to those in *O. oratoria*, presenting a relatively simpler evolutionary pattern. Specifically, *IR1018* and *IR1021* cluster within the decapod-conserved tuning *IR* group. In *M. nipponense*, a similar close relationship between co-receptor *IRs* and *iGluRs* is observed. Moreover, *M. nipponense* possesses the largest number of tuning *IRs*, which are also distributed among the three branches of tuning *IRs*. Notably, among conserved tuning *IRs*, *M. nipponense* lacks *IR75*, *IR1018*, and *IR1069*. These results reveal distinct evolutionary patterns for these genes, indicating their conservation and functional diversification during crustacean evolution.

### 3.4. Motif, Gene Structure, and Domain Prediction Analysis of the IR Gene Family

**Figure 3:**
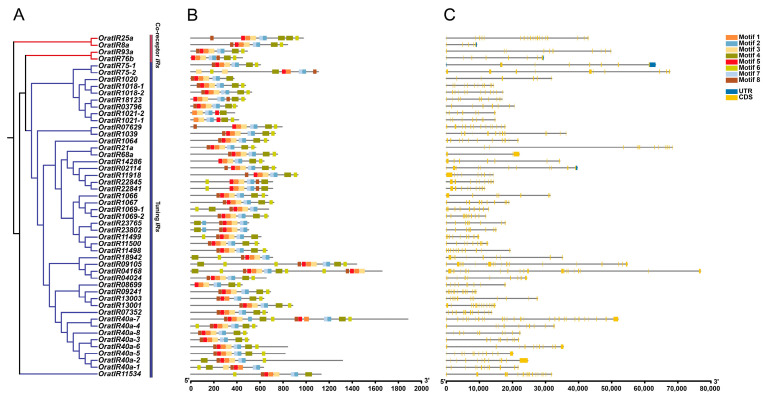
Phylogenetic relationships, conserved motifs, and gene structures of the *OratIRs*. (**A**) Phylogenetic relationships of the *OratIRs*. Red denotes *iGluRs*, and blue denotes *IRs*. (**B**) Conserved motifs of the *OratIRs*. Motifs 1 to 8 are marked with different colors. (**C**) Gene structures of the *OratIRs*. Blue boxes indicate UTR regions, yellow boxes indicate CDS regions, and black lines represent intron regions.

To explore the diversity of *OratIR* protein structures, we analyzed 50 *IR* protein sequences using the MEME online tool. The analysis of conserved motifs within the *IR* gene family members of *O. oratoria* (Figure 3B) identified a total of eight conserved motifs, designated as motif 1 through motif 8, with detailed sequence information provided in Figure S1. The number and distribution of motifs across each group of *IR* proteins are generally similar, ranging from a minimum of 6 to a maximum of 15 motifs. Among these eight motifs, motif 1, motif 2, motif 4, and motif 5 are uniformly distributed; all members share these four motifs in common. Consequently, they represent typical domains of *IRs*, suggesting that they may have analogous functions. Gene structure analysis (Figure 3C) showed that the total number of *OratIRs*’ exons ranged from 3 to 28, while the number of introns varied between 2 and 27. The co-receptor *IRs* exhibit exon counts ranging from 5 to 19 and intron counts from 4 to 18, whereas tuning *IRs* display exon numbers ranging from 3 to 28 and intron numbers varying from 2 to 27. Notably, both maximum and minimum values for exons and introns are found in tuning *IRs*. Overall, substantial differences exist in the exon and intron structures of *IRs* within *O. oratoria*.

**Figure 4:**
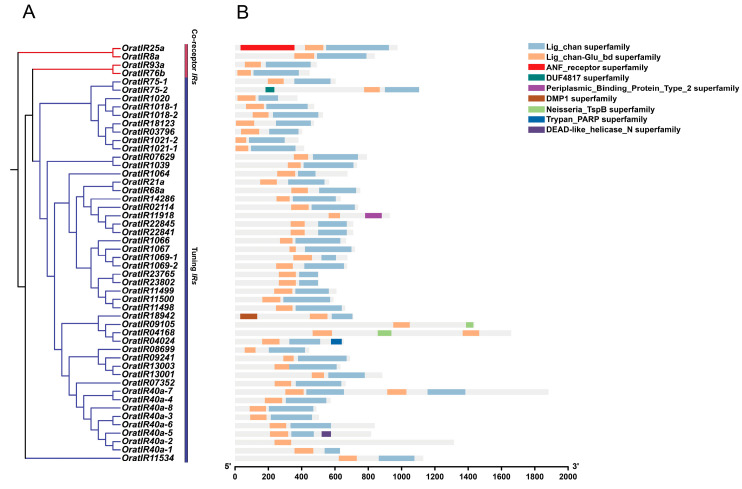
Phylogenetic relationships and protein domain analysis of the *OratIRs*. (**A**) Phylogenetic relationships of the *OratIRs*. Red denotes *iGluRs*, and blue denotes *IRs*. (**B**) Protein domain of the *OratIRs*. Different colored boxes represent various protein domain superfamilies.

Protein domain information for the *IR* gene family members of *O. oratoria* was obtained using the CD-search tool on the NCBI website (Figure 4B). The results indicate that both the co-receptor *IRs* and tuning *IRs* subfamilies within the *IR* gene family of *O. oratoria* exhibit highly conserved structural characteristics. All four members of the co-receptor *IRs* possess both Lig_chan (Ligand-gated ion channel, PF00060) and Lig_chan-Glu_bd (Ligand-binding site for L-glutamate and glycine, PF10613). Collectively, these domains constitute the core functional module of the *IRs*, playing essential roles in the construction and regulation of ion channels as well as in the specific recognition of ligands. The Lig_chan superfamily comprises four transmembrane regions (M1–M4) present in *iGluRs* and NMDA receptors. The NMDA receptor, a subtype of *iGluRs*, operates through an activation mechanism that relies on the synergistic action of two ligands—L-glutamate and glycine—playing a critical regulatory role in synaptic signaling and neural plasticity. The Lig_chan-Glu_bd superfamily functions as a specific binding site for L-glutamate and glycine, situated within the S1 domain of the receptor. Consequently, the Lig_chan-Glu_bd domain represents a critical functional region for *iGluRs* family members (such as NMDA receptors) to bind ligands effectively, sense them, and activate ion channels. This mechanism enables *IRs* to respond precisely to external chemical signals. Additionally, *OratIR25a* also includes members from the ANF_receptor superfamily (Receptor family ligand binding region, PF01094). Tuning *IRs* demonstrate a high degree of similarity to co-receptor *IRs* in their domain compositions. Most conserved tuning *IRs* members concurrently possess the Lig_chan superfamily and the Lig_chan-Glu_bd superfamily. However, it is noteworthy that, among the arthropod-conserved tuning *IRs*, *OratIR40a-2*, as one of the few exceptions, contains only the Lig_chan-Glu_bd superfamily. *OratIR40a-5*, in addition to the Lig_chan superfamily and Lig_chan-Glu_bd superfamily, also includes the DEAD-like_helicase_N superfamily. Similarly, *OratIR75-2* exhibits a unique domain combination that encompasses the Lig_chan superfamily and Lig_chan-Glu_bd superfamily, but also the DUF4817 superfamily. In other tuning *IRs*, *OratIR04024* not only contains the Lig_chan superfamily and the Lig_chan-Glu_bd superfamily, but also shows the presence of the Trypan_PARP superfamily. Likewise, *OratIR18942*, in addition to the Lig_chan superfamily and Lig_chan-Glu_bd superfamily, also has the DMP1 superfamily. *OratIR09105* and *OratIR04168* only contain the Lig_chan-Glu_bd superfamily and the Neisseria_TspB superfamily. Furthermore, *OratIR11918* is another member with a unique combination of domains, containing only the Lig_chan-Glu_bd superfamily and the Periplasmic Binding_Protein_Type_2 superfamily.

The secondary structure of *OratIR* proteins comprises alpha helix (Hh), extended strands (Ee), and random coils (Cc), with the highest proportion being random coils, which range from 35.73% to 76.73%. This is followed by alpha helices, accounting for 12.24% to 47.13%, while the proportion of extended strands is the smallest, ranging from 11.03% to 30.96% (Table 4). Utilizing known gene segments of *OratIR* proteins, homology modeling of the tertiary structure of *OratIR* proteins was conducted using SWISS-MODEL. The results (Figure S2) reveal notable differences among the 50 *OratIR* proteins; variability in their spatial structures underlies their functional distinctions. Consequently, variations in spatial structures among *OratIR* proteins dictate their functional differences.

### 3.5. Protein Interaction Network of Genes Related to IRs

**Figure 5:**
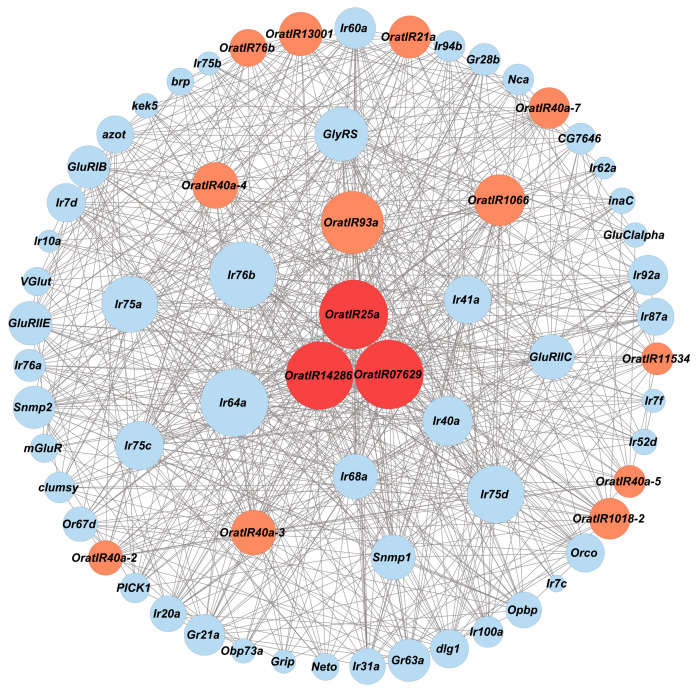
Protein–protein interactions of *OratIRs*. Circles with red or orange represent *IRs* in *O. oratoria*, blue circles represent *IRs* in *Drosophila*, and the size of the circles indicates the number of connected nodes.

To investigate and analyze protein–protein interactions and infer the potential functions of the *IR* gene family in sensing and regulation, we examined the *OratIR* protein interaction network using the String tool, with *Drosophila* serving as a control model organism from the arthropods. The analysis revealed that a total of 15 *OratIR* proteins are involved in protein interactions, as illustrated in Figure 5. Notably, *OratIR25a*, *OratIR07629*, and *OratIR14286* exhibit the highest number of interactions, serving as pivotal nodes in the network. Furthermore, all three demonstrate strong interaction relationships with *OratIR93a*, *Ir40a*, *Ir64a*, and *Ir76b*.

Based on the functional annotations derived from the String database (Tables S4 and S5), *OratIR25a*, *OratIR93a*, *OratIR14286*, and *Ir40a* participate in processes such as chemical stimulus detection, ion transmembrane transport, and functions related to the nervous system. Additionally, they exhibit activities related to inorganic molecular entity transmembrane transporter activity and ion channel activity. Specifically, *OratIR25a* is primarily involved in responding to environmental stimuli and regulating circadian rhythms through mechanisms that include temperature response, temperature compensation of the circadian clock, and entrainment of the circadian clock. *OratIR07629* serves as an auxiliary receptor for ligand-specific *IRs* that predominantly detect a variety of organic acids while playing a crucial role in biological processes such as responses to acetate, chlorate, and inorganic substances. *Ir64a* serves as a broadly specific acid sensor that primarily detects acidic substances within the environment, and is involved in responses to acidic pH levels as well as reactions to chlorate. *Ir76b* plays a significant role in the chemical sensing of amines and salts, including the detection of chemical stimuli involved in the sensory perception of salty taste, with salty taste receptor activity and sodium ion transmembrane transporter activity. With amine compounds specifically, *Ir76b* is implicated in responses to organonitrogen compounds.

### 3.6. FISH Detection of OratIR8a and OratIR75-1

**Figure 6:**
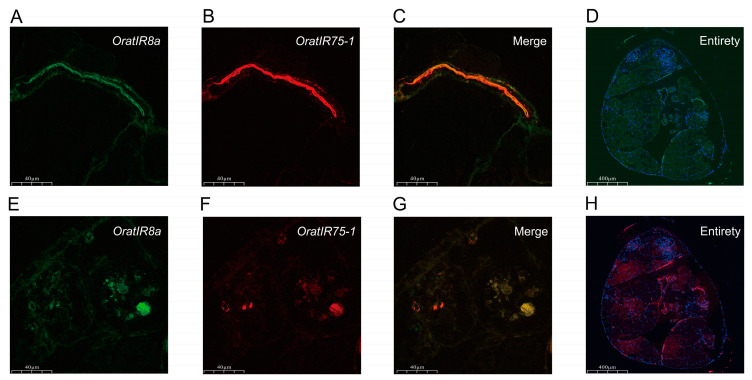
The expression of *OratIR8a* and *OratIR75-1* in the antennae of *O. oratoria*. (**A**,**E**) Green fluorescence shows labeling with the *OratIR8a* probe, scale bar: 40 µm; (**B**,**F**) red fluorescence shows labeling with the *OratIR75-1* probe, scale bar: 40 µm; (**C**,**G**) co-labeling with the *OratIR8a* probe and the *OratIR75-1* probe indicated by yellow-orange fluorescence, scale bar: 40 µm; (**D**) the entirety of the antennae co-labeled with DAPI (blue) and the *OratIR8a* probe (green), scale bar: 400 µm; (**H**) the entirety of the antennae co-labeled with DAPI (blue) and the *OratIR75-1* probe (red), scale bar: 400 µm.

*OratIR8a* serves as a co-receptor for ligand-specific *IRs*, playing a critical role in mediating the binding of specific ligands to their receptors, while *OratIR75-1*, an important member of the expanded gene family, has been demonstrated to exhibit strong responsiveness to various acidic compounds [13,24,58,59,60]. Based on their unique molecular characteristics and potential functional significance, we employed fluorescent in situ hybridization (FISH) to systematically investigate the localization and co-expression patterns of these two genes in the antennae of *O. oratoria*. In the sections examined, the signal from the *OratIR8a* probe is represented by green fluorescence (FAM), while that from the *OratIR75-1* probe is indicated by red fluorescence (Cy3). The results (Figure 6) demonstrate that, following fluorescent in situ hybridization, fluorescein-labeled genes are detectable within the antennae, with robust expression signals observed for both *OratIR8a* and *OratIR75-1* in the antennae. Furthermore, both receptors are expressed in identical locations within the antennae, indicating a clear co-expression phenomenon.

### 3.7. Expression Levels of Five OratIRs in O. oratoria Muscle and Tentacles

**Figure 7:**
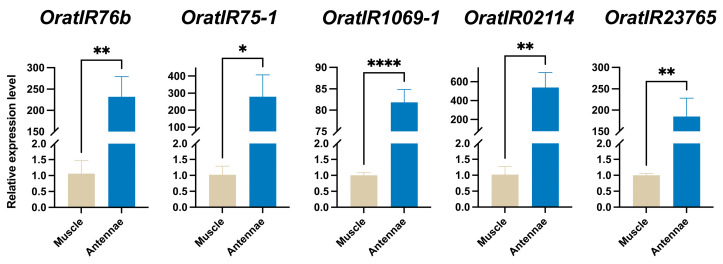
Relative expression levels of *OratIRs* in muscle and antennae tissues. *, *p* < 0.05; **, *p* < 0.01; ****, *p* < 0.0001.

In this study, the expression levels of five genes from the *OratIR* family (including *OratIR76b*, *OratIR75-1*, *OratIR1069-1*, *OratIR02114*, and *OratIR23765*) in the muscle and antennal tissues of *O. oratoria* were quantified using RT-qPCR. The results demonstrated that the expression levels of all five *OratIRs* were significantly higher (*p* < 0.05) in antennal tissue compared to muscle tissue (Figure 7). This finding further confirms that *OratIRs* are highly expressed in the antennae of *O. oratoria* and may play a critical role in its olfactory evolution.

## 4. Discussion

Crustaceans employ their sensitive and intricate olfactory systems to detect critical chemical signals in their external environment, thereby regulating a variety of behavioral activities including foraging, mating, egg-laying, and predator avoidance [12,61,62]. Olfactory-related receptors are essential for signal transduction and represent some of the most important components of chemical communication [63]. Consequently, investigating these receptors is essential for elucidating the olfactory coding mechanisms in crustaceans. *IRs* are an identified class of olfactory-related receptors within the *iGluR* family; they were first characterized in the model organism *Drosophila*, in which substantial progress has been made in understanding their functions [14]. With the ongoing advancement and maturation of molecular biology technologies, an increasing number of various types and quantities of *IRs* have been identified. These include those from species such as *Daphnia pulex* [15], *Panulirus argus*, *Homarus americanus*, *Procambarus clarkii*, *Callinectes sapidus* [31,35,64], *Coenobita clypeatus* [30,65], and *Scylla paramamosain* [66]. In this study, we identified the *IR* genes of *O. oratoria*, *L. vannamei*, and *M. nipponense* at the whole-genome level. Our findings reveal that *O. oratoria* possesses 50 *IR* genes, while *L. vannamei* has 28, and *M. nipponense* exhibits 74 *IR* genes. There are obvious differences in the number of *IR* genes among different species. These differences likely stem from gene duplication or loss events, selective pressures imposed by diverse ecological niches, and functional constraints, which result in some gene subtypes being more conserved than others.

The amino acid length of the *OratIR* gene family ranges from 375 to 1882 aa, with a theoretical pI between 4.0 and 9.4, and the acidic proteins constitute 68% of this family, suggesting that *OratIR* proteins are enriched in acidic amino acids and possess the potential to function effectively in acidic subcellular environments. This finding is analogous to observations made regarding *Gynaephora qinghaiensis* [67]. In the study, the markedly higher proportion of hydrophilic proteins (63%) among *OratIRs* indicates their strong hydrophilicity. As receptors for sensing water-soluble compounds, this hydrophilic structure enables them to interact effectively with non-volatile compounds dissolved in water [15]. This characteristic aligns with the functional traits of the aquatic ancestors of protostomes, which preferentially recognize water-soluble hydrophilic acids and amines through *IRs* [24,68]. Therefore, the differences in amino acid length, pI, and other characteristics among different subfamilies of *OratIRs* may be closely related to the diversity of their gene functions. Subcellular localization prediction results indicate that most *OratIR* genes are predominantly located on the plasma membrane, which is consistent with the characteristics of *IRs* as variants within the *iGluR* subfamily. *iGluRs* detect external chemical signals by regulating cation flow across the plasma membrane, thereby influencing cells’ intrinsic physiological states [13,15]. It can be preliminarily inferred that the 35 *IR* genes situated on the plasma membrane serve as crucial components involved in recognizing and transmitting odor molecules, and those located within the endoplasmic reticulum, mitochondria, and vacuoles may be associated with material transport and synthesis.

Through chromosome localization analysis, *OratIRs* were found to be distributed across multiple chromosomes. This study identified six groups of tandemly repeated genes on chromosomes 11, 14, 24, and 30, as well as on scaffold 87. Notably, among these, the gene duplication events of *IR40a* and *IR75* have also been similarly reported in arthropods [31,35,60,69,70,71]. Additionally, *IR75* was found to be responsive to various acidic compounds [15,60]. Gene duplication serves as a crucial evolutionary mechanism, supplying new genetic material that can lead to novel gene functions through processes such as subfunctionalization or neofunctionalization [72,73]. These expanded gene families may facilitate the identification of similar chemical substances and enhance their differentiation [16]. Using FISH, we found that *OratIR75-1* and *OratIR8a* are co-expressed in the antenna tissue of *O. oratoria*; similar findings in *Agrotis segetum*, where *AsegIR75p/q* and *AsegIR8a* were localized in the basal sensilla or tricho sensilla [60], suggest that *IR* genes exhibit polymorphic receptor localization in arthropods.

Through phylogenetic analysis of the *IR* and *iGluR* gene families in *O. oratoria*, the constructed intraspecific phylogenetic tree was divided into two branches. Co-receptor *IRs* show a close relationship with *iGluRs*. Specifically, one branch clusters *IR25a*, *IR8a*, and non-NMDA *iGluRs* together. This finding aligns with previous studies suggesting that *IRs* may share a common evolutionary origin with AMPA or Kainate receptors within the non-NMDA *iGluRs* [15,16]. On the other hand, the tuning *IRs* subfamily, which makes up a relatively large proportion, can be further split into three branches, including various conserved tuning *IRs* reported in previous studies [31,35]. Interspecies phylogenetic analysis revealed that the phylogenetic tree is also divided into two branches. Among different species, the gene subtypes of *IRs* and *iGluRs* cluster separately. The separate clustering of genes of each subfamily of *iGluRs* (including NMDA *iGluRs* and non-NMDA *iGluRs*) and *IRs* (including co-receptor *IRs* and tuning *IRs*) indicates that the divergence of the *IR* gene family occurred before the divergence of *O. oratoria*, *L. vannamei*, and *M. nipponense*.

Gene structure analysis demonstrated substantial variations in the number and positioning of introns and exons. The maximum and minimum numbers of exons and introns are both observed in tuning *IRs*, which display greater diversity, while co-receptor *IRs* remain relatively conserved, akin to *Heliconius butterflies* [74]. This architectural diversity may drive gene family evolution, endowing genes with novel functions and enhancing crustacean adaptability. Through the analysis of conserved structural domains, tuning *IRs* show a high degree of similarity in domain assembly with co-receptor *IRs*. Most members contain the Lig_chan and Lig_chan-Glu_bd superfamilies structure, aligning with characteristics typical of the conserved ligand-gated ion channel domains shared by *iGluRs* and *IRs*, thus maintaining their core functions [15]. Among these, the Lig_chan-Glu_bd superfamily possesses the ability to facilitate precise ligand binding and trigger conformational changes in the ion channel upon ligand interaction, thereby activating the channel [18,75]. However, some members exhibit structural diversity; for instance, *OratIR25a* possesses an additional ANF_receptor structural domain involved in assembling *iGluR* subunits and binding cofactors [76]. *OratIR11918* contains PBP2, which can efficiently capture specific chemical ligands and synergistically interact with transmembrane transport complexes to facilitate substance transport across membranes [18,77]. Results from secondary structure prediction reveal that *OratIR* proteins are predominantly characterized by random coils and alpha helices, akin to *G. qinghaiensis* [67]. The random coil conformation exhibits inherent flexibility, while the spatial structures of alpha helices demonstrate enhanced stability [78]. Furthermore, variations in tertiary structure among different proteins indicate that both similarities and differences in spatial configuration may play an important role in contributing to functional diversity.

Protein–protein interaction analysis revealed that *OratIR25a*, *OratIR07629*, and *OratIR14286* serve as pivotal nodes in protein interactions, all engaging with the proteins *OratIR93a*, *Ir40a*, *Ir64a*, and *Ir76b*. Among these genes, *IR25a* functions as a core receptor that collaborates with multiple partners. Research conducted in *Drosophila* has demonstrated that the roles of *IRs* extend beyond olfaction to include sensory processes such as temperature sensing, humidity detection, and taste perception [79]; within the protein–protein interaction (PPI) network, *OratIR14286* corresponds to the *IR21a* gene in *Drosophila*, and the protein it encodes is a component of the antennal neural sensory system, participating in the environmental temperature response. It has been demonstrated that *IR25a* works in concert with *IR21a* and *IR93a* to modulate the physiological response of *Drosophila* to cold temperatures [17,26]. On the other hand, *OratIR07629* corresponds to the *IR8a* gene in *Drosophila*. The *IR8a* gene encodes an ionotropic receptor family member that functions as an auxiliary receptor for detecting organic acids. Studies have shown that *IR64a* and *IR8a* form a functional ligand-gated ion channel that mediates *Drosophila*’s response to acidic chemicals (e.g., acetic acid, propionic acid, butyric acid). Specifically, while *IR64a* primarily detects acidic odors, it is regulated by *IR8a*, which ensures proper protein levels and transport for optimal function. Both receptors are co-expressed within specific neurons of *Drosophila*, where they jointly modulate physiological and behavioral responses to acidic environments [58,80]. *IR93a* is co-expressed with both *IR25a* and *IR40a* in the sacculus neurons of the antennae and contributes substantially to humidity sensing (hygrosensation). In particular, *IR40a* plays a primary role in detecting humidity by discerning dry air from variations in moisture levels [27,28,81]. Additionally, *IR76b* acts as a co-receptor alongside *IR25a* for perceiving acidic substances while also being involved in detecting other chemicals such as low salt concentrations and amino acids. Furthermore, these receptor complexes play an essential role in guiding *Drosophila*’s selection of acidic environments for oviposition [82,83,84,85]. In summary, *OratIR25a*, *OratIR07629*, and *OratIR14286* play essential roles in coordinating the organism’s perception of temperature fluctuations, humidity changes, and organic acids, as well as its behavioral responses.

## 5. Conclusions

This study explores the *Ionotropic Receptors* (*IRs*), a less-researched chemosensory gene family in stomatopods. Based on the whole-genome data of *O. oratoria*, 50 *IR* genes were identified and classified into two subfamilies: co-receptor *IRs* and tuning *IRs*. Members of the same subfamily exhibited similar conserved motifs and domains. Through comparative analysis, the expansion and evolutionary diversification of *IR* genes in *O. oratoria*, *L. vannamei*, and *M. nipponense* were revealed. Phylogenetic analysis indicated that co-receptor *IRs* were highly conserved among the three crustaceans. However, many tandem repeat genes were found in the tuning *IRs* subfamily, among which *OratIR40a* was the most abundant in number. The *OratIRs* show strong hydrophilicity, enabling them to interact with non-volatile compounds in water. Through analysis of the protein interaction network, it was found that *OratIR25a*, *OratIR07629*, and *OratIR14286* play an important role in the perception of temperature and acidic odors. *OratIR75-1* was co-expressed with *OratIR8a* in the antenna tissue of *O. oratoria*, as detected by FISH. These findings deepen our understanding of the chemical sensory system in *O. oratoria* and provide a valuable reference for further exploring the evolution and function of *IR* genes.

## Figures and Tables

**Table 1:** Primers used for fluorescence in situ hybridization (FISH) experiments.

Probe Name	Primer Sequence (5′~3′)	Usage
*OratIR8a*	CCGTCACTGATTGGTGAAGACAGG	*OratIR8a* in situ hybridization probe utilized FAM (488) fluorochrome for visualization, with the excitation light-emitting green fluorescence.
	CTTGATCCAGCTCTGCTTCATTGC GAGCTGTTTGGGTCATGTCCCTGC CCAGTGACCACAAGAGCAGCAAC GGTTGTTCAGCAAAGGGCTCACC	
*OratIR75-1*	GGCATCACTCAAAGACTTCGGAGC	*OratIR75-1* in situ hybridization probe utilized Cy3 fluorochrome for visualization, with the excitation light-emitting red fluorescence.
	CGGAGGTGGAGCCCAGTAAGGTTC	
	GGTGTCGGGAAGTCGACGTACTG	
	GAGGTTACTCGTGTAGAAGGCCAG	
	CGTAGGTCCAGGAGAACATCACTG	

Each probe is composed of a mixture of five oligonucleotides and is a multi-sequence mixed probe.

**Table 2:** Primers used for qPCR assays.

Primer Name	Forward Primer (5′~3′)	Reverse Primer (5′~3′)
*OratIR76b*	GTATGGTGGGAATGGTCAA	TCGTAGTAGGCGTGGGTG
*OratIR75-1*	TCAACTACGGAGCAGGAA	GAGAAGAAGGAAGAGGATGG
*OratIR1069-1*	GTGGTGGTGATGATGATGA	CGTCCTTCTCCTCCTCTT
*OratIR02114*	GATGCTGCTGATGCTACTA	TGTGAAGGAGGAGGTTGT
*OratIR23765*	AGACTGACCACGACAACGG	GGAGTATAAGACCCAAGCACC
*β-actin*	ATCGTTCGTGACATTAAGGA	CAAGGAATGAAGGCTGGAA

**Table 3:** Physicochemical properties and subcellular localization of the *OratIRs*.

Gene Name	Amino Acid Length (aa)	Molecular Weights (kDa)	Isoelectric Point (pI)	Instability Index	Aliphatic Index	Grand Average of Hydropathicity (GRAVY)	Subcellular Localization
*OratIR8a*	839	93.47	4.9	39.84	82.3	−0.182	Plasma membrane
*OratIR25a*	976	109.73	4.88	39.43	83.05	−0.178	Plasma membrane
*OratIR93a*	491	56.15	5.91	38.98	81.14	−0.117	Endoplasmic reticulum
*OratIR76b*	448	52.3	9.08	37.11	86.14	−0.16	Golgi
*OratIR21a*	565	62.27	6.57	42.43	105.88	0.188	Plasma membrane
*OratIR40a-1*	631	70.84	5.91	50.29	92.69	−0.208	Plasma membrane
*OratIR40a-2*	1315	151.95	4.98	76.62	80.87	−0.119	Endoplasmic reticulum
*OratIR40a-3*	504	56.18	5.47	38.54	88.81	0.061	Plasma membrane
*OratIR40a-4*	574	64.06	5.57	36.1	104.23	0.207	Plasma membrane
*OratIR40a-5*	818	93.00	7.56	41.92	99.66	−0.087	Plasma membrane
*OratIR40a-6*	839	92.98	4.00	27.98	83.03	−0.553	Mitochondrial
*OratIR40a-7*	1882	219.30	4.22	87.04	70.65	−0.902	Extracellular
*OratIR40a-8*	489	54.30	8.61	38.49	96.67	0.065	Golgi
*OratIR68a*	752	85.18	8.37	44.96	91.9	−0.042	Plasma membrane
*OratIR75-1*	604	68.58	9.4	52.13	85.91	−0.273	Golgi
*OratIR75-2*	1107	127.23	9.32	56.41	73.21	−0.584	Plasma membrane
*OratIR1018-1*	475	53.49	7.63	43.45	97.77	0.176	Plasma membrane
*OratIR1018-2*	528	58.59	5.35	47.3	93.41	0.082	Plasma membrane
*OratIR1020*	375	42.32	6.38	31.37	95.76	0.113	Plasma membrane
*OratIR1021-1*	415	46.31	8.18	42.58	91.33	0.028	Plasma membrane
*OratIR1021-2*	381	42.88	5.65	45.99	89.55	0.021	Plasma membrane
*OratIR1039*	733	83.05	6.79	41.43	94.13	−0.048	Plasma membrane
*OratIR1064*	675	75.20	6.41	37.38	91.41	−0.035	Endoplasmic reticulum
*OratIR1066*	667	76.17	8.32	40.3	95.13	0.01	Plasma membrane
*OratIR1067*	720	82.01	8.01	44.37	91.51	−0.137	Endoplasmic reticulum
*OratIR1069-1*	675	76.12	4.21	30.81	75.24	−0.577	Golgi
*OratIR1069-2*	673	75.48	5.92	35.6	98.96	−0.02	Plasma membrane
*OratIR02114*	739	83.72	7.27	48.36	99.01	0.017	Plasma membrane
*OratIR03796*	403	44.04	8.63	33.91	106.92	0.229	Plasma membrane
*OratIR04024*	653	73.52	7.67	38.57	95.93	−0.076	Plasma membrane
*OratIR04168*	1658	184.00	6.68	48.18	87.22	−0.222	Plasma membrane
*OratIR07352*	665	73.96	5.97	39.36	96.03	0.070	Vacuolar
*OratIR07629*	792	88.69	6.36	42.34	87.37	−0.093	Plasma membrane
*OratIR08699*	446	49.18	6.39	36.01	90.47	−0.017	Plasma membrane
*OratIR09105*	1438	159.58	7.42	49.87	87.20	−0.248	Plasma membrane
*OratIR09241*	691	77.18	8.97	35.37	100.85	0.045	Mitochondrial
*OratIR11498*	662	75.03	5.79	43.36	92.51	−0.026	Plasma membrane
*OratIR11499*	609	68.88	8.11	39	85.81	−0.057	Plasma membrane
*OratIR11500*	591	66.83	5.71	34.15	87.11	−0.052	Endoplasmic reticulum
*OratIR11534*	1130	127.38	4.77	72.91	68.18	−0.756	Plasma membrane
*OratIR11918*	929	105.23	6.62	43.4	89.89	−0.174	Golgi
*OratIR13001*	884	97.37	5.62	46.85	91.07	−0.147	Plasma membrane
*OratIR13003*	633	71.53	6.14	39.38	102.56	0.089	Plasma membrane
*OratIR14286*	635	73.69	5.42	43.88	89.94	0.026	Endoplasmic reticulum
*OratIR18123*	474	53.44	6.54	45.6	90.46	−0.185	Plasma membrane
*OratIR18942*	710	79.4	6.67	50.78	90.01	−0.183	Plasma membrane
*OratIR22841*	710	80.31	5.31	47.53	91.46	−0.083	Plasma membrane
*OratIR22845*	710	80.27	5.39	47.6	91.61	−0.081	Plasma membrane
*OratIR23765*	502	56.35	6.24	35.34	90.28	−0.034	Plasma membrane
*OratIR23802*	502	56.3	6.08	35.85	90.08	−0.027	Plasma membrane

**Table 4:** Summary of two-dimensional structures of the *OratIRs*.

Gene Name	Gene ID	Alpha Helix (Hh)	Extended Strand (Ee)	Random Coil (Cc)
*OratIR8a*	*Orat_gene07298*	318/37.90%	163/19.43%	358/42.67%
*OratIR25a*	*Orat_gene16135*	349/35.76%	198/20.29%	429/43.95%
*OratIR93a*	*Orat_gene16136*	106/21.59%	152/30.96%	233/47.45%
*OratIR76b*	*Orat_gene21236*	130/29.02%	98/21.88%	220/49.11%
*OratIR21a*	*Orat_gene17732*	185/32.74%	99/17.52%	281/49.73%
*OratIR40a-1*	*Orat_gene07733*	242/38.35%	106/16.80%	283/44.85%
*OratIR40a-2*	*Orat_gene21360*	161/12.24%	145/11.03%	1009/76.73%
*OratIR40a-3*	*Orat_gene21361*	151/29.96%	121/24.01%	232/46.03%
*OratIR40a-4*	*Orat_gene21362*	202/35.19%	122/21.25%	250/43.55%
*OratIR40a-5*	*Orat_gene21363*	270/33.01%	182/22.25%	366/44.74%
*OratIR40a-6*	*Orat_gene21364*	234/27.89%	163/19.43%	442/52.68%
*OratIR40a-7*	*Orat_gene21367*	887/47.13%	264/14.03%	731/38.84%
*OratIR40a-8*	*Orat_gene21368*	178/36.40%	94/19.22%	217/44.38%
*OratIR68a*	*Orat_gene00368*	237/31.52%	159/21.14%	356/47.34%
*OratIR75-1*	*Orat_gene17760*	251/41.56%	70/11.59%	283/46.85%
*OratIR75-2*	*Orat_gene17762*	337/30.44%	195/17.62%	575/51.94%
*OratIR1018-1*	*Orat_gene29704*	161/33.89%	117/24.63%	197/41.47%
*OratIR1018-2*	*Orat_gene29705*	179/33.90%	117/22.16%	232/43.94%
*OratIR1020*	*Orat_gene06678*	168/44.80%	73/19.47%	134/35.73%
*OratIR1021-1*	*Orat_gene20793*	114/27.47%	100/24.10%	201/48.43%
*OratIR1021-2*	*Orat_gene20797*	137/35.96%	75/19.69%	169/44.36%
*OratIR1039*	*Orat_gene07724*	237/32.33%	138/18.83%	358/48.84%
*OratIR1064*	*Orat_gene06913*	207/30.67%	131/19.41%	337/49.93%
*OratIR1066*	*Orat_gene18414*	241/36.13%	151/22.64%	275/41.23%
*OratIR1067*	*Orat_gene09232*	239/33.19%	166/23.06%	315/43.75%
*OratIR1069-1*	*Orat_gene09184*	127/18.81%	164/24.30%	384/56.89%
*OratIR1069-2*	*Orat_gene09189*	254/37.74%	122/18.13%	297/44.13%
*OratIR02114*	*Orat_gene02114*	237/32.07%	155/20.97%	347/46.96%
*OratIR03796*	*Orat_gene03796*	151/37.47%	74/18.36%	178/44.17%
*OratIR04024*	*Orat_gene04024*	182/27.87%	156/23.89%	315/48.24%
*OratIR04168*	*Orat_gene04168*	413/24.91%	345/20.81%	900/54.28%
*OratIR07352*	*Orat_gene07352*	265/39.85%	118/17.74%	282/42.41%
*OratIR07629*	*Orat_gene07629*	261/32.95%	174/21.97%	357/45.08%
*OratIR08699*	*Orat_gene08699*	156/34.98%	81/18.16%	209/46.86%
*OratIR09105*	*Orat_gene09105*	345/23.99%	304/21.14%	789/54.87%
*OratIR09241*	*Orat_gene09241*	261/37.77%	122/17.66%	308/44.57%
*OratIR11498*	*Orat_gene11498*	189/28.55%	128/19.34%	345/52.11%
*OratIR11499*	*Orat_gene11499*	132/21.67%	155/25.45%	322/52.87%
*OratIR11500*	*Orat_gene11500*	173/29.27%	119/20.14%	299/50.59%
*OratIR11534*	*Orat_gene11534*	446/39.47%	182/16.11%	502/44.42%
*OratIR11918*	*Orat_gene11918*	260/27.99%	157/16.90%	512/55.11%
*OratIR13001*	*Orat_gene13001*	221/25.00%	193/21.83%	470/53.17%
*OratIR13003*	*Orat_gene13003*	267/42.18%	111/17.54%	255/40.28%
*OratIR14286*	*Orat_gene14286*	204/32.13%	140/22.05%	291/45.83%
*OratIR18123*	*Orat_gene18123*	166/35.02%	117/24.68%	191/40.30%
*OratIR18942*	*Orat_gene18942*	146/20.56%	169/23.80%	395/55.63%
*OratIR22841*	*Orat_gene22841*	213/30.00%	161/22.68%	336/47.32%
*OratIR22845*	*Orat_gene22845*	216/30.42%	158/22.25%	336/47.32%
*OratIR23765*	*Orat_gene23765*	114/22.71%	131/26.10%	257/51.20%
*OratIR23802*	*Orat_gene23802*	114/22.71%	132/26.29%	256/51.00%

## Data Availability

The genomic datasets used in this study are publicly available from the National Center for Biotechnology Information (NCBI) database under the following accession numbers: *Oratosquilla oratoria* (GCA_046742065.1), *Litopenaeus vannamei* (GCA_003789085.1), and *Macrobrachium nipponense* (GCA_015104395.1).

## Supplementary Materials

The following supporting information can be downloaded at: https://www.mdpi.com/article/10.3390/ani15060852/s1, Supplementary Figure S1. Sequence logos for the conserved motifs of the *OratIRs*, Supplementary Figure S2. The three-dimensional structure prediction of the *OratIRs*; Supplementary Table S1. IR/*iGluR* Protein Sequence IDs, Supplementary Table S2. Sequence list of the *IR* genes identified from *O. oratoria*, *L. vannamei*, and *M. nipponense* genomes, Supplementary Table S3. Location of the *OratIRs* in tandem duplications, Supplementary Table S4. Protein interactions of *OratIRs* with *Drosophila* as a model, Supplementary Table S5. String interaction annotations of *OratIRs* with *Drosophila* as a model.
