# Supplementary material for: Genome-Wide Identification and Evolutionary Analysis of Ionotropic Receptors Gene Family: Insights into Olfaction Ability Evolution and Antennal Expression Patterns in Oratosquilla oratoria
Source: Animals (Basel). 2025 Mar 16;15(6):852. doi: 10.3390/ani15060852 (PMC11939437; doi:10.3390/ani15060852)
Supplement: Supplementary file 1 [file animals-15-00852-s001.zip › Table S3.pdf]

Table S3. Location of the *OratIRs* in tandem duplications.

Gene Name	Gene ID	Chromosome No.	Start site (bp)	End site (bp)
OratIR1069-1	*Orat_gene09184*	CHR11	45411242	45424032
OratIR1069-2	*Orat_gene09189*	CHR11	45455944	45467894
OratIR11498	*Orat_gene11498*	CHR14	26735248	26754566
OratIR11499	*Orat_gene11499*	CHR14	26764618	26774438
OratIR11500	*Orat_gene11500*	CHR14	26780058	26792584
OratIR75-1	*Orat_gene17760*	CHR24	17107652	17170920
OratIR75-2	*Orat_gene17762*	CHR24	17218846	17286432
OratIR40a-2	*Orat_gene21360*	CHR30	42124770	42149409
OratIR40a-3	*Orat_gene21361*	CHR30	42154890	42176799
OratIR40a-4	*Orat_gene21362*	CHR30	42205063	42237756
OratIR40a-5	*Orat_gene21363*	CHR30	42238915	42259108
OratIR40a-6	*Orat_gene21364*	CHR30	42268943	42304355
OratIR40a-7	*Orat_gene21367*	CHR30	42434220	42486182
OratIR40a-8	*Orat_gene21368*	CHR30	42510607	42532949
OratIR1018-1	*Orat_gene29704*	SCAFFOLD87	385925	400190
OratIR1018-2	*Orat_gene29705*	SCAFFOLD87	408066	425210
