# Supplementary figures and images for: Genome-Wide Identification and Evolutionary Analysis of Ionotropic Receptors Gene Family: Insights into Olfaction Ability Evolution and Antennal Expression Patterns in Oratosquilla oratoria
Source: Animals (Basel). 2025 Mar 16;15(6):852. doi: 10.3390/ani15060852 (PMC11939437; doi:10.3390/ani15060852)

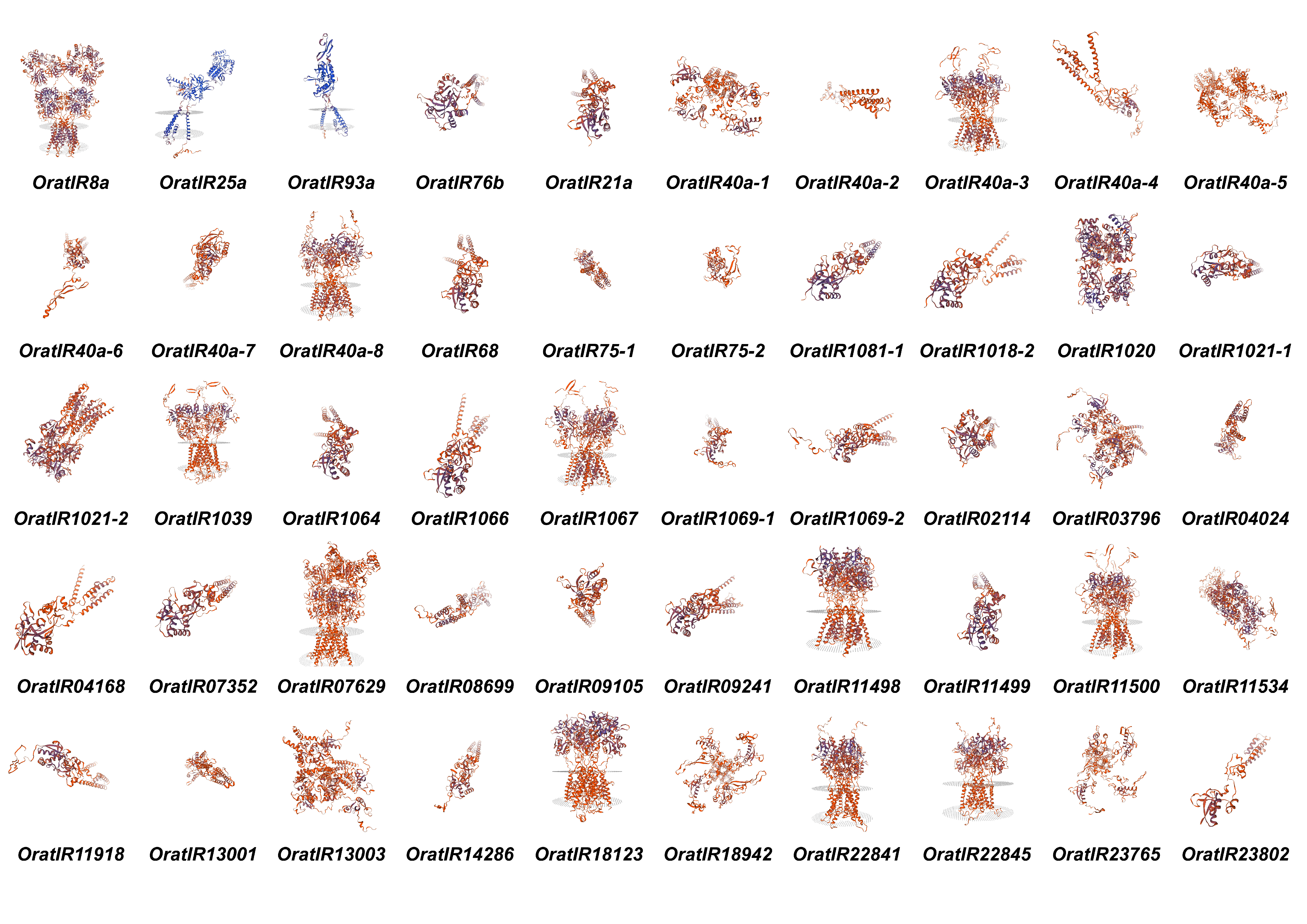

Supplement: Supplementary file 1 [file animals-15-00852-s001.zip › Figure S2.jpg]
